# Posttransplant Metabolic Syndrome

**DOI:** 10.1155/2012/891516

**Published:** 2012-11-27

**Authors:** M. Shadab Siddiqui, Richard K. Sterling

**Affiliations:** ^1^Division of Gastroenterology, Hepatology and Nutrition, Virginia Commonwealth University, Richmond, VA 23298-0341, USA; ^2^Section of Hepatology, Division of Gastroenterology, Virginia Commonwealth University, Richmond, VA 23298-0341, USA

## Abstract

Metabolic syndrome (MS) is a cluster of metabolic derangements associated with insulin resistance and an increased risk of cardiovascular mortality. MS has become a major health concern worldwide and is considered to be the etiology of the current epidemic of diabetes and cardiovascular disease. In addition to cardiovascular disease, the presence of MS is also closely associated with other comorbidities including nonalcoholic fatty liver disease (NAFLD). The prevalence of MS in patients with cirrhosis and end-stage liver disease is not well established and difficult to ascertain. Following liver transplant, the prevalence of MS is estimated to be 44–58%. The main factors associated with posttransplant MS are posttransplant diabetes, obesity, dyslipidemia, and hypertension. In addition to developing NAFLD, posttransplant MS is associated with increased cardiovascular mortality that is 2.5 times that of the age- and sex-matched individuals. Additionally, the presence of posttransplant MS has been associated with rapid progression to fibrosis in individuals transplanted for HCV cirrhosis. There is an urgent need for well-designed prospective studies to fully delineate the natural history and risk factors associated with posttransplant MS. Until then, early recognition, prevention, and treatment of its components are vital in improving outcomes in liver transplant recipients.

## 1. Introduction

Metabolic syndrome (MS) is a cluster of metabolic derangements associated with insulin resistance and an increased risk of cardiovascular mortality. According to the Adult Treatment Panel III definition (Table 1), MS is defined as the presence of dyslipidemia, obesity, glucose intolerance, and hypertension [1]. 

Metabolic syndrome has become a major health concern worldwide and is considered to be the etiology of the current epidemic of diabetes and cardiovascular disease. According to the Framingham study, MS alone can predict at least 25% of all new onset cardiovascular disease. The Third National Health and Nutrition Examination Survey (NHANES) in 1999-2000 estimated the age-adjusted prevalence of MS in the adult US population to be 24% and is projected to increase further with the increasing prevalence of obesity and diabetes [2]. In addition to cardiovascular disease, the presence of MS is also closely associated with other comorbidities including nonalcoholic fatty liver disease (NAFLD), cholelithiasis, polycystic ovary disease, and obstructive sleep apnea. The aim of this paper is to discuss the importance and impact of metabolic syndrome and its component in liver-transplant recipients. Although only evidence that pertains of liver transplantation will be discussed, similar metabolic complications have been observed in patients undergoing other solid organ transplants.

## 2. Nonalcoholic Fatty Liver Disease (NAFLD) and Metabolic Syndrome

NAFLD is considered to be the hepatic manifestation of MS and is defined as the presence of >5% deposition of triglycerides in the liver in the absence of significant alcohol consumption (<20–40 g/day for women and 40–80 g/day for men). Up to 90% of patients with NAFLD have at least 1 feature of the MS, with 33% having all components of the MS. The reported prevalence of NAFLD varies widely depending on the population studied and modality used to make the diagnosis but is estimated to be 6.3% to 33% in the general population. Nonalcoholic steatohepatitis (NASH), the most aggressive phenotype of NAFLD, is characterized by the presence of hepatocyte injury, cytologic ballooning, and inflammation and has an estimated prevalence of 3–5%. Unlike NAFLD, NASH is associated with a decreased patient survival compared to the general population due to the increased cardiovascular risk. Additionally, the presence of NASH is associated with the increased risk of progression to cirrhosis and a need for liver transplantation. A recent analysis of the Scientific Registry of Transplant Recipients (SRTRs) confirmed that the NASH as an indication for liver transplant increased over 7-fold from 2001 to 2009, while no other indication for liver transplantation increased over the same time period [3]. Already being the 3rd most common indication for liver transplant, it is poised to surpass HCV as the leading indication for liver transplant in the near future due to an unparalleled increase in features of MS.

## 3. Posttransplant Metabolic Syndrome (PTMS)

The prevalence of MS in patients with cirrhosis and end-stage liver disease is not well established and difficult to be ascertained due to changes usually associated with end-stage liver disease. The low systemic vascular resistance and low lipid levels associated with chronic liver disease reduce the likelihood that patients with cirrhosis would meet ATP III criteria. Presence of ascites in cirrhotic patients can further confound the diagnosis of obesity thereby, making the diagnosis of MS in patients with cirrhosis difficult. However, there are data to suggest that the prevalence of MS in cirrhosis and end-stage liver disease likely varies with the etiology of liver disease and is likely higher in patients with cryptogenic or NASH cirrhosis [4]. 

Liver transplantation is an effective therapy for chronic end-stage liver disease. Improvement in surgical techniques, management of infectious complications, and immunosuppression have led to excellent long-term survival rates in liver-transplant recipients. Consequently, death related to metabolic consequences (cardiovascular disease and malignancies) is becoming increasingly important as hepatic etiologies of late post-liver-transplant death become less common. 

**Figure 1:**
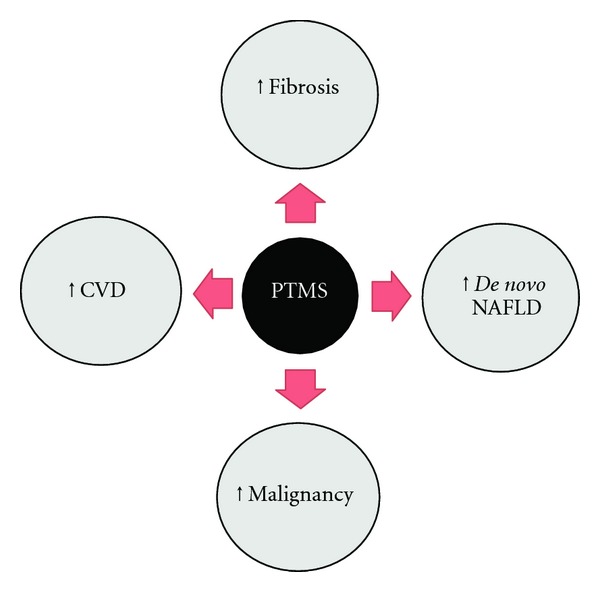
Complications of Posttransplant Metabolic Syndrome (PTMS). CVD = cardiovascular disease, NAFLD = nonalcoholic fatty liver disease.

The limited number of studies evaluating the incidence of PTMS in liver-transplant recipients has considerable variability in the reported data due to differing definitions of MS used [5]. Recent studies estimate the prevalence of PTMS to be 44–58% in liver-transplant recipients and is associated with increased cardiovascular mortality (Table 2) [6, 7]. The relative risk of cardiovascular death in liver-transplant recipients is 2.5 times that of the age- and sex-matched individuals (Figure 1) [8]. Additionally, the presence of PTMS has been associated with a rapid progression to fibrosis in individuals transplanted for HCV cirrhosis [9]. This risk is even greater (30% versus 8%, *P* < 0.01) in individuals with PTMS compared to those without it [6]. Since PTMS can affect 1 out of 2 transplant recipients and can account for up to 42% cardiovascular disease related mortality, its impact on liver-transplant recipients is immense [8, 10, 11]. The main factors associated with PTMS are posttransplant diabetes, obesity, dyslipidemia, and hypertension (Table 2), which can result in posttransplant NAFLD.

## 4. Posttransplant Diabetes Mellitus (PTDM)

Up to 60–80% of patients with cirrhosis may have glucose intolerance and 20% may develop DM; it is due to profound peripheral resistance, decreased glycogen synthesis, and impaired glucose oxidation [12, 13]. Unfortunately, up to third of patients will remain diabetic after liver transplantation [14, 15]. Posttransplant diabetes mellitus confers a twofold increased risk of cardiovascular and liver related deaths in liver-transplant recipients [10]. Presence of diabetes can also have detrimental impact on graft survival. PTDM is associated with increased advanced graft fibrosis, late onset hepatic artery thrombosis, recurrent or *de novo* fatty liver disease, and acute and chronic rejection [14, 16–18]. Additionally, the mortality and morbidity in liver-transplant recipients is higher even when posttransplant diabetes is transient [17, 19]. 

Earlier studies in liver-transplant recipients reported the prevalence of PTDM 1 year after transplant from 13–27% using the fasting plasma glucose of 140 mg/dL as the diagnostic criteria [5, 17, 20, 21]. However, using the more recent diagnostic criteria of fasting plasma glucose of 126 mg/dL, Laryea et al. reported the prevalence of PTDM to be 61% [6]. In one study, 80% of new onset diabetes (NOD) occurred within the first month after liver transplant and only a small minority (12%) developed NOD after the 1st year after transplant [14]. 

Although limited by retrospective data and small cohorts, factors associated with PTDM include HCV and alcohol related cirrhosis as indications for transplant (*P* < 0.05), pretransplant DM (OR = 24.4, *P* < 0.01), male gender, HCV infection, and steroid use (*P* < 0.05) [7, 16, 21, 22]. High doses of corticosteroids are an integral part of the early immunosuppressive regiments in many transplant centers. Corticosteroids lead to insulin resistance and diabetes by decreasing insulin production, increasing gluconeogenesis, and decreasing peripheral glucose utilization [23]. Decreasing the dose of prednisone from 10 mg to 5 mg per day reduced the prevalence of PTDM (*P* = 0.045) [5]. Similarly, reducing the daily dose of prednisone from 13 ± 4 mg at 1 year to 2 ± 4 mg at 3 years leads to a 20% reduction in the prevalence of PTDM [20]. In a recent meta-analysis, the relative risk of diabetes (RR = 0.29, *P* < 0.001) was attenuated when corticosteroids were replaced by another immunosuppressive agent [24]. These effects appear to be transient as the prevalence of diabetes in post-liver-transplant recipients reverts to that of patients on steroid-free regiments once corticosteroids are discontinued [25]. 

Calcineurin inhibitors, cyclosporine (CsA) and Tacrolimus (FK506), are associated with an increased risk of PTDM, with the incidence possibly being higher with the use of Tacrolimus [7, 17, 21, 26]. The increased risk of posttransplant diabetes associated with Tacrolimus (RR 1.38, CI 1.01–1.86) use compared to CsA in liver transplant recipient was confirmed in a recent Cochrane review [27]. The calcineurin inhibitors exert their diabetogenic effects by inhibiting pancreatic *β*-cell ability and diminishing insulin synthesis and secretion [28]. Calcineurin inhibitors also reduce peripheral glucose utilization leading to peripheral insulin resistance. 

Finally the data regarding the impact of Sirolimus, an mTOR inhibitor, on posttransplant diabetes is conflicting. Chronic mTOR inhibition has been associated with reduced pancreatic *β*-cell mass, reduced hepatic insulin clearance, and increased gluconeogenesis, thereby causing insulin resistance [29]. On the other hand, activation of the mTOR pathway via glucose leads to the inhibition of insulin receptor substrate-2 (IRS-2), increase *β*-cell apoptosis and insulin resistance [30]. Therefore, the impact of Sirolimus on PTDM remains unclear. 

## 5. Posttransplant Obesity

Obesity (body mass index (BMI) >30 kg/m^2^) is a common sequela of liver transplantation affecting 21–42% liver-transplant recipient [5, 31–33]. Risk factors for post-OLT obesity include donor BMI, absence of acute rejection, and steroid use [31]. Additionally, patients who are overweight or obese before transplant will likely remain overweight or obese after transplant. Furthermore, patients who were not obese at the time of transplant, 16% became obese at 1 year and 26% at 3 years [32]. 

Well-known side effect of corticosteroid use is weight gain and truncal obesity. Although corticosteroids have been traditionally associated with greater posttransplant weight gain, available literature suggests otherwise [32, 33]. This is likely due to a reduction in dosing and duration of steroid use as well as emergence of steroid-free or steroid-sparing immunosuppressive regiments. Cyclosporine compared to Tacrolimus was associated with an additional 2.3 kg gain 1 year after transplant [26]. However, these differences were not significant 3 years after transplant. 

## 6. Posttransplant Dyslipidemia

Dyslipidemia is common after transplant affecting 45–69% of liver-transplant recipients [7, 19, 34–37]. One study reported the pretransplant prevalence of dyslipidemia rose from 8% to 66% after liver transplant in patients who were followed for over 14 months [35]. More specifically, prevalence of hypercholesterolemia and hypertriglyceridemia increased from 2.9% and 18.2% before transplant to 15.3% and 70% after transplant, respectively [38]. The prevalence of low HDL after transplantation is reported to be 48–52% [6, 39]. 

Risk factors of hypercholesterolemia in liver-transplant recipients include pretransplant hypercholesterolemia, cyclosporine, and corticosteroid use [35, 39]. Predictors of posttransplant hypertriglyceridemia include cirrhosis resulting from HCV, HBV, alcohol, cryptogenic cirrhosis and posttransplant renal insufficiency [35]. Although, long-term therapy with corticosteroids can result in dyslipidemia, it is unclear how corticosteroids impact long-term dyslipidemia in posttransplant population [40]. Corticosteroids can lead to dyslipidemia by increasing the hepatic production of lipids, increased production of very low-density lipoprotein (VLDL) cholesterol, and decreased hepatic LDL reuptake. 

Although both calcineurin inhibitors are also associated with posttransplant dyslipidemia and the relationship between posttransplant dyslipidemia and cyclosporine is more robust. Cyclosporine inhibits hepatic bile acid 26-hydroxylase, which is thought to decrease reverse cholesterol transport or transport of cholesterol into bile and its subsequent elimination into the intestines [41]. Additionally, cyclosporine binds to LDL receptor and thereby decreases LDL-cholesterol uptake [15, 39]. Conversion from cyclosporine to Tacrolimus results in improvement in both serum cholesterol and triglyceride levels but has no impact on HDL cholesterol [42, 43]. 

Sirolimus is associated with posttransplant hypertriglyceridemia and elevated serum LDL cholesterol. Sirolimus alters the insulin-signaling pathway by increasing adipose tissue lipase activity, decreasing lipoprotein lipase activity which results in increased hepatic triglyceride synthesis, increased secretion of VLDL and thereby causing hypertriglyceridemia [44]. Additionally, cyclosporine and Sirolimus work synergistically to promote dyslipidemia and should be avoided in patients with underlying dyslipidemia [44, 45]. This synergistic effect is not seen with Sirolimus and Tacrolimus. 

## 7. Posttransplant Hypertension

Since patients with cirrhosis, particularly decompensated cirrhosis, have decreased systemic vascular resistance, hypertension is only present in a small minority of patients before transplant but can affect 62–69% of liver-transplant recipients [5, 9, 45, 46]. Post-transplant hypertension may result from increased renal vasoconstriction and impaired sodium excretion induced by cyclosporine use and may occur less frequently with Tacrolimus use [23, 46]. Patients treated specifically with cyclosporine, the prevalence of hypertension was 58–82%, while the incidence of posttransplant hypertension was 31–38% in patients treated with Tacrolimus [26, 47, 48]. In animal models, cyclosporine generates interstitial fibrosis without any significant decrease in renal blood flow or structural arteriolar lesion, through early macrophage influx and increased TGF-*β* expression. Additionally, since cyclosporine-induced ischemia and tubulointerstitial injury can occur independently, preventing renal injury with CsA altogether could be difficult [49]. Data regarding the use of Sirolimus and posttransplant hypertension is still evolving and no definitive statements can be made. 

## 8. Posttransplant Nonalcoholic Fatty Liver Disease

Nonalcoholic fatty liver disease is closely associated with features of metabolic syndrome and likely represents the hepatic manifestation of the metabolic syndrome. *De novo* NALFD after transplant was initially reported in a retrospective study where 75% of patients transplanted for NASH had fatty infiltration of the graft and 38% developed NASH [50]. In patients transplanted for cryptogenic cirrhosis, time-dependent risk of developing allograft steatosis was 100% by five years [51]. Additionally, 25% of patients transplanted for alcoholic and cholestatic liver disease developed fatty liver disease. The risk of developing *de novo* NAFLD after liver transplant is associated with pretransplant obesity, a higher BMI at the time of the last biopsy, and a higher post-transplant BMI [52, 53]. Patients with greater than 10% increase in pretransplant BMI had a significantly higher risk of developing *de novo* NAFLD compared to those without weight gain. Unfortunately, the natural history of posttransplant *de novo* fatty liver disease is currently unknown but it is possible that post-transplant fatty liver disease contributes to the increased cardiovascular mortality since NAFLD is an independent risk factor CVD in noncirrhotic patients. Well-designed prospective trials are needed to confirm this assertion. 

## 9. Conclusion

Metabolic syndrome and its components are common in liver-transplant recipients and associated with increased cardiovascular disease, fibrosis, *de novo* NAFLD after transplant (Figure 1), and decreased patient and graft survival. There is an urgent need for well-designed prospective studies to fully delineate the natural history and risk factors associated with PTMS. In the interim, early recognition, prevention, and treatment of components of PTMS are vital in improving outcomes in liver-transplant recipients. 

## Figures and Tables

**Table 1:** National cholesterol education program: Adult Treatment Panel III criteria for metabolic syndrome.

Abdominal obesity	Waist circumference
	>102 cm in men
	>88 cm in women

Glucose intolerance	Fasting plasma glucose ≥ 100 mg/dL (5.6 mmol/L)

Hypertension	Blood pressure ≥ 130/85 mmHg or on therapy for hypertension

Hypertriglyceridemia	Serum triglycerides ≥ 150 mg/dL or on therapy for hypertriglyceridemia

Low HDL-C	Serum HDL-C < 40 mg/dL (1 mmol/L) in men Serum HDL-C < 50 mg/dL (1.3 mmol/L) in women

**Table 2:** Prevalence of Posttransplant Metabolic Syndrome  (PTMS) and its components.

PTMS	45–58%
Obesity	21–43%
Hypertension	62–69%
Hypertriglyceridemia	66–85%
Low HDL-C	48–52%
Posttransplant *de novo* fatty liver disease	18–40% (100% recurrence in patients transplanted for cryptogenic cirrhosis at 5 years)
